# Reserve-related activities and MRI metrics in multiple sclerosis patients and healthy controls: an observational study

**DOI:** 10.1186/s12883-016-0624-1

**Published:** 2016-07-19

**Authors:** Carolyn E. Schwartz, Michael G. Dwyer, Ralph Benedict, Bianca Weinstock-Guttman, Niels P. Bergsland, Jei Li, Murali Ramanathan, Robert Zivadinov

**Affiliations:** DeltaQuest Foundation, Inc, 31 Mitchell Road, Concord, MA 01742 USA; Departments of Medicine and Orthopaedic Surgery, Tufts University Medical School, Boston, MA USA; Buffalo Neuroimaging Analysis Center, Department of Neurology, School of Medicine and Biomedical Sciences, State University of New York, Buffalo, NY USA; Department of Biomedical Informatics, University of Buffalo, State University of New York, Buffalo, NY USA; Department of Neurology, School of Medicine and Biomedical Sciences, University of Buffalo, State University of New York, Buffalo, NY USA; Istituto Di Ricovero e Cura a Carattere Scientifico, “S.Maria Nascente”, Don Gnocchi Foundation, Milan, Italy; Department of Electronics, Information and Bioengineering, Politecnico di Milano, Milan, Italy; Department of Biostatistics, University of Massachusetts, Amherst, MA USA; Department of Pharmaceutical Sciences, School of Medicine and Biomedical Sciences, State University of New York, Buffalo, NY USA; MR Imaging Clinical Translational Research Center, School of Medicine and Biomedical Sciences, University at Buffalo, State University of New York, Buffalo, NY USA

## Abstract

**Background:**

To examine whether past and current reserve-related activities make the brain less susceptible to MS pathology (i.e., lesions or disease-related atrophy).

**Methods:**

This secondary analysis of a cohort study included 276 healthy controls (HC), and 65 clinically isolated syndrome (CIS), 352 relapsing-remitting MS (RR) and 109 secondary- progressive MS (SPMS) patients. Past reserve-related activities comprised educational and occupational attainment. Current reserve-related activities comprised strenuous and non-strenuous activities. MRI was performed on 3 T scanner. Regression and non-parametric analysis examined relationships between MRI metrics and reserve-related activities.

**Results:**

Multivariate models (HC as referent) revealed significant interactions in predicting strenuous reserve-related activities with chronic lesion burden (for CIS), brain- (for RR & SPMS), subcortical- (for CIS, RR, & SPMS) and amygdala- (for RR) volumes. Maximal Lifetime Brain Growth was higher for RR patients who engaged in running before and after diagnosis, rather than only before or never. Residual Brain Volume was higher in RR patients who did weights-exercise before and after diagnosis, as compared to only before.

**Conclusions:**

Reserve-related activities are related to brain health cross-sectionally in all MS subgroups, and longitudinally in RR patients. Consistent with reserve theory, RR patients who maintained strenuous activities had higher Maximal Lifetime Brain Growth and Residual Brain Volume. The study’s limitations are discussed, including the potential for recall bias and design limitations that preclude causal inference.

**Electronic supplementary material:**

The online version of this article (doi:10.1186/s12883-016-0624-1) contains supplementary material, which is available to authorized users.

## Background

A growing body of research suggests that reserve is a key concept underlying resilience to neurological disease [1]. Conceptualized as a buffer between measurable disease pathology and actual level of function, reserve can be studied by investigating brain maintenance [2]. Maintenance theories emphasize neuro-protective mechanisms. Current conceptualizations of reserve posit that past enrichment activities and current stimulating leisure activities play an important role in helping an individual to build and maintain reserve [3]. These “reserve-related activities” in theory bring both history and current activities into focus. In recent work, these activities are operationalized in terms of past activities (e.g., educational and occupational attainment) and current activities that are considered stimulating [4–7].

Recent research has documented a protective effect of stimulating leisure activities [6] in a broad range of disability domains in multiple sclerosis (MS) [4], such as physical, creative, intellectual, spiritual, and cultural enrichment. While both past and current reserve-related activities had notable cross-sectional and longitudinal associations with health and well-being, the current activities trumped past activities in explaining variance in health outcomes [4, 8]. Reserve-related activities provide a fundamental resource to the individual [9], and are associated with better health habits, maintaining employment, and living independently [8]. Distinct from different aspects of insight into one’s condition [10], high reserve-related activity engagement is associated with underlying cognitive appraisal processes that focus on the positive and more controllable aspects of their condition [11].

Longitudinal data supported a significant buffering effect of reserve, such that high-reserve individuals showed slower disability progression over six years of semi-annual follow-up [4]. These findings support the idea that symptom burden worsens as damage to the brain accumulates, and when reserve is exhausted, the progressive stage of the disease begins [12]. To date, however, very little research has addressed how these reserve-related activities relate to MRI metrics of brain health. Sumowski and colleagues reported that the negative effect of brain atrophy on information processing efficiency was attenuated at higher levels of reserve operationalized as education, such that MS subjects with higher levels of education were able to better withstand MS neuropathology without suffering cognitive impairment [13]. Further, early life enriching leisure activities were found to be more protective against memory deficits than cognitive inefficiency [14]. A retrospective analysis of neuroimaging data suggested that recreational activities protect against brain atrophy’s detrimental influence on cognition [15].

All of these prior studies suffered from small samples sizes and limited operationalizations of reserve or reserve-related activities. We thus sought to use a large and rich data set to test the *hypothesis* that engaging in past or current reserve-related activities will make the brain less susceptible to MS pathology in the forms of lesions or disease-related atrophy. The study data set includes a large sample with MRI data, including a substantial reference group of healthy controls who provide important referent information for the multivariate analyses. It includes a novel measure estimating residual brain volume, and includes data on exercise that captured events and pattern changes before and after MS diagnosis. Building on an initial descriptive study of differences in reserve-related activities between healthy individuals and people with MS, the present work examines how these reserve-related activities relate to MRI metrics of Maximal Lifetime Brain Growth and Residual Brain Volume.

## Methods

### Sample

This secondary analysis utilized data from an ongoing prospective study of cardiovascular, environmental and genetic risk factors in MS at the MS Center of the State University of New York at Buffalo which enrolled over 1,000 subjects with clinically isolated syndrome (CIS) [16–18], MS, healthy controls, and other neurologic diseases [19, 20]. For the purpose of this analysis, we focused on comparing people with CIS and MS to healthy controls. The sample included 65 (8 %) people with CIS; 352 (44 %) people with relapsing-remitting MS (RRMS); and 109 people (14 %) with secondary progressive MS (SPMS). There were also 276 (34 %) age- and gender-matched healthy controls. The inclusion criteria for this sub-analysis were presence of sufficient questionnaire data to obtain past and current reserve-related activity estimates. The exclusion criteria were presence of relapse and steroid treatment in the 30 days preceding study - entry- MRI for CIS and MS patients, pre-existing medical conditions known to be associated with brain pathology (cerebrovascular disease, positive history of alcohol abuse), and pregnancy. Healthy controls needed to meet the health-screen requirements, and had to have a normal physical and neurological examination. They were recruited from hospital personnel, or were respondents to a local advertisement.

### Procedure

All subjects were assessed with a structured epidemiologic questionnaire administered in-person by a trained interviewer unaware of the subjects’ disease status [16]. This procedure was aimed at minimizing missing data.

### Patient consents

This study was approved by the local State University of New York Buffalo Health Sciences Institutional Review Board (HSIRB #NEU2490109A) and written informed consent was obtained from all subjects.

### Measures

#### Patient-reported

In addition to demographic characteristics, the questionnaire queried physically strenuous (i.e., exercise) activities as well as strenuous and non-strenuous activities (e.g., hobbies or other pastimes), similar to those included in questionnaires investigating reserve [8, 21]. Based on the psychometric analyses described in [5], we created a derived measure of reserve-related activities comprised of three summary scores. These summary scores represented composite scores comprised of average scores for each factor score resulting from a principal components factor analysis. The first summary score was *Past Reserve-related Activities*, which summarized educational and occupational attainment. The second was *Current Strenuous Reserve-related Activities*, which included items for currently endorsed contact sports, aerobics, swimming, and wrestling. The third was *Current Non-Strenuous Reserve-related Activities*, which included items for currently endorsed reading, browsing the internet, meditation, and doing puzzles. These summary scores ranged from 2–10, 0–5.4, and 0–13.5, respectively. These scores reflect frequency of endorsement on an ordinal scale. Table 1 shows the descriptive statistics by group.

**Table 1:** Study participant demographics

Variable	HC	CIS	RRMS	SPMS	Test Statistic	*P*-value	*P*-valueª
N	276	65	352	109			
Gender: % female	60.87	61.54	64.77	61.47	2.97	0.81	
Mean Age (sd)	46.93 (15.76)	39.38 (10.99)	44.17 (10.75)	53.91 (8.69)	23.62	0.00	***
Mean Age at Diagnosis (sd)	NA	36.25 (10.92)	34.90 (9.41)	36.21 (10.09)	1.03	0.36	
Median EDSS (IQR)	NA	1.50 (1–2)	2 (3–1.5)	6 (5–6.5)	24.64	0.00	***
Diease Duration: mean no. years (sd)	NA	5.65 (3.32)	13.1 (7.11)	21.85 (9.10)	83.61	0.00	***
Range	NA	3–20	2–41	5–45			
Mean Body Mass Index (sd)	27.31 (5.70)	27.14 (5.86)	27.40 (5.93)	26.10 (6.01)	1.35	0.26	
Employment Status %					201.79	0.00	***
Full-time	48.22	61.9	45.38	11.32			
Part-time	15.81	11.11	12.14	10.38			
Homemaker	3.16	7.94	3.47	4.72			
Student	7.91	4.76	2.02	0			
Unemployed	8.3	6.35	6.65	5.66			
Retired	13.83	3.17	6.65	24.53			
Disabled	0	3.17	21.97	41.51			
Other	2.77	1.59	1.73	1.89			
Education %					30.85	0.01	*
High school not completed	1.99	1.59	3.78	7.55			
Graduated high school	15.54	12.7	17.15	21.7			
Some college/Associate/Technical Degree	34.66	38.1	36.05	33.96			
Bachelor’s degree	25.9	30.16	23.26	19.81			
Graduate/Post-graduate	21.91	17.46	19.77	16.98			
Race %					39.52	0.00	***
Caucasian	83.59	92.06	91.91	95.28			
Hispanic/Latino	1.56	95.24	2.02	0.94			
Black/African-American	8.59	98.41	4.91	2.83			
Asian	3.91	100	0.58	0			
American Indian/Alaska Native	0.39	0	0	0			
Other	1.95	0	0.58	0.94			
Past reserve-related Activities: Mean (sd)	6.60 (0.13)	6.44 (1.76)	6.27 (2.06)	5.75 (2.04)	4.39	0.00	***
Range	2–10	3–10	2–10	2–10			
Strenuous reserve-related Activities: Mean (sd)	1.61 (0.078)	0.70 (0.67)	0.64 (0.70)	0.36 (0.41)	76.73	0.00	***
Range	0–5.42	0–2.83	0–3.23	0–1.8			
Non-Strenuous reserve-related Activities : Mean(sd)	5.80 (0.16)	5.18 (2.47)	5.36 (2.62)	5.53 (2.84)	1.64	0.18	
Range	0.5–12.67	0–12	0–13.5	0–12.83			

ª*HC* Healthy Control, *CIS* Clinically Isolated Syndrome, *RRMS* Relapsing Remitting MS, *SPMS* Secondary Progressive MS, The F-Statistics and P-values shown are from tests for differences between disease group for the given variable

* *p <* 0.05; ** *p <* 0.01; *** *p <* 0.001

#### Neuroimaging

Full details of the scan protocol have been previously reported [22]. Briefly, fluid-attenuated inversion recovery, T1 spin echo (pre and post gadolinium injection) and 3D-T1 images were acquired on a 3 T GE Signa Excite HD 12.0 Twin Speed 8-channel scanner (General Electric, Milwaukee, WI, USA). T2, T1, and gadolinium-enhancing-lesion volumes (LV) were calculated using a reliable semi-automated edge detection contouring/thresholding technique using JIM software. We used SIENAX to obtain normalized tissue volumes of the brain, gray and white matter as well as the lateral ventricles. The 3D-T1 images were preprocessed using a lesion filling technique to reduce the impact of white matter lesions on volumetric segmentations [23]. Tissue volumes for the bilateral thalamus, caudate, putamen, globus pallidus, hippocampus, amygdala and nucleus accumbens were calculated using FMRIB’s FIRST, a model-based segmentation and registration tool [24].

### Statistical analysis

#### Descriptive analyses

Descriptive statistics of demographic variables were computed by disease group, and linear models were used to compare group values for each demographic variable.

#### Maximal lifetime brain growth

As previously reported [25], and consistent with other researchers [14], we used the SIENAX scaling factor as a surrogate for intracranial volume (ICV) to determine Maximal Lifetime Brain Growth, since ICV is a reliable estimate of Maximal Lifetime Brain Growth. This metric reflects the maximum brain volume attained in the individual’s lifetime, prior to brain atrophy associated with aging and disease progression. In terms of reserve theory [3], this metric would be considered *brain reserve*. This variable was transformed into gender-specific z-scores in order to adjust for the expected difference in ICV [25].

#### Residual brain volume

We used a regression technique to produce a measure of residual brain volume [26]. Briefly, we regressed normalized brain parenchymal volume (NBPV) against T2 lesion volume, age, EDSS, MS disease duration, and gender to produce per-subject measures of expected brain volume based on demographic and overall MS disease severity. Then, we subtracted this expected NBPV from the observed NBPV. Thus, negative values represent more brain atrophy than expected, and positive values represent less. In terms of reserve theory [3], this metric would be related to *modifiable reserve*.

#### Regression analyses

##### Data reduction

See supplementary text for full description of data reduction analyses done prior to inferential statistical analysis. These analyses yielded the following MRI variables for analysis: Chronic Brain Burden (T1 and T2 lesion volume), Active Brain Burden (Contrast-enhanced lesion volume), Brain Volume, and Subcortical Gray Matter Volume. They yielded past, current strenuous, and current non-strenuous reserve-building activities.

##### Multivariable analysis of MRI structural metrics

To examine the relationship between MRI metrics, disease-group, and reserve-related activities, we computed regression models for each of the three reserve-related activities summary scores: past, current strenuous and current non-strenuous. The model included relevant covariates (age and gender); MRI composite(s) (chronic lesion burden, active lesion burden, brain volume, and subcortical gray matter volume in separate models); dummy variables for MS disease group (CIS, RRMS, and SPMS), with healthy controls as the comparison/referent group; and finally the variables of particular interest in these analyses: the interaction terms between MS dummy variables and MRI metrics. These interaction terms test if there is a differential relationship between MRI and reserve metrics by MS disease group. The Type I error rate for each model was 5 %.

##### Investigating change in strenuous activities and brain structure in relapsing-remitting group

We then created a variable that summarized change in strenuous activities from before and after MS diagnosis in relapsing-remitting patients. Separate change variables were created for each of the strenuous activities (aerobics, running, walking, weights, yoga, and taichi); and medians were plotted by group for the above-described two derived MRI metrics of specific relevance to reserve: Maximal Lifetime Brain Growth and Residual Brain Volume.

The distributions of strenuous activities by change group were visualized using box plots, and then non-parametric tests were used to compare the distributions, first in an omnibus Kruskal-Wallis test and then in pairwise Wilcoxon tests [27]. This set of analyses was only implemented in the RR subgroup of MS patients for several reasons. First, this was the largest subgroup of MS patients in the sample (*n =* 352), and thus the analyses would have better statistical power to detect relevant differences. Second, reserve theory would predict that reserve is more intact in RR patients [12] and thus the hypothesized relationships between reserve-related activities, Maximal Lifetime Brain Growth, and current reserve would be clinically relevant.

All analyses were implemented using Stata 13 [28] and R Studio™ version 0.98.1103 [29].

## Results

### Sample

Table 1 provides the descriptive statistics on the study sample, as well as comparisons among groups. The sample included 276 healthy controls, 65 people with CIS, 352 with RR, and 109 with SPMS. The majority of the sample was female, and the mean age in the various subsamples ranged from 39–54 years. Mean age at diagnosis in the various subsamples ranged from 35 to 36 years, and median Expanded Disability Status Scale (EDSS) score in the various subsamples ranged from 1.5 to 6.0. The mean disease duration ranged from 5.7 to 21.9 years (range 2–45 years). The sample had a body- mass- index consistent with being overweight. The majority of the healthy controls, CIS and RR samples were employed either full- or part-time, but the employed SPMS cohort constituted only 22 % of the sample. The majority of the sample had some college education or greater, and the sample was predominantly Caucasian. The subsamples included in this study differed by age, EDSS, disease duration, employment status, education, and race.

### Reserve metrics descriptive statistics and correlations by group

Mean scores and score ranges for the various reserve-related activities are shown in Table 1. The lowest score ranges were in the current strenuous reserve-related activities (range 0–5.4), and healthy controls reported the highest scores in these activities, followed by CIS, RR, and SPMS patients, respectively (*p <* 0.001). There were no differences between subsamples in engagement in non-strenuous activities, but group differences were found in past-reserve building activities, with SPMS patients reported the lowest scores (*p <* 0.01).

### Multivariable analysis of MRI structural metrics

Mean scores and standard deviations for the various MRI metrics are provided in Additional file 1: Table S1. Results of the regression models suggest that there are no significant predictors of past reserve-related activities or non-strenuous reserve-related activities (Table 2a and c). Consequently, the regression models explained almost no variance in past and non-strenuous reserve-related activities. In contrast, the models predicting strenuous reserve-related activities suggested that there was a differential positive association for CIS with chronic lesion burden; a differential negative association for RR and SPMS with brain volume; and a differential negative association with CIS, RR, and SPMS with subcortical gray matter volume, and with RR and amygdala volume (Table 2b). Further, the models explained 10 %, 9 %, 28 %, and 29 % of the variance in strenuous reserve-related activities, after adjusting for the number of predictors (Table 2a-c). There were no significant group-by-active lesion interactions predicting strenuous reserve-related activities.

**Table 2:** a-c Regression models predicting reserve-related activities^a^

Dependent Variable:											
2a Past Reserve Building Activities				2b Strenuous Current reserve-related Activities				2c Non-Strenuous Current reserve-related Activities			
Predictors	β	t	*p*	Predictors	β	t	*p*	Predictors	β	t	*p*
Chronic lesion burden models											
Age	0.01	0.13	0.90	Age	–0.19	–3.94	0.00	Age	–0.05	–0.94	0.35
Gender	–0.02	–0.42	0.68	Gender	–0.07	–1.72	0.09	Gender	–0.05	–1.07	0.29
Chronic Burden	–0.31	–1.25	0.21	Chronic Burden	–0.34	–1.46	0.15	Chronic Burden	0.20	0.82	0.41
CIS	0.01	0.08	0.94	CIS	–0.37	–5.04	0.00	CIS	–0.03	–0.43	0.67
RR	–0.03	–0.24	0.81	RR	–0.51	–5.33	0.00	RR	0.09	0.87	0.38
PMS	–0.14	–1.37	0.17	PMS	–0.51	–5.42	0.00	PMS	0.13	1.32	0.19
Chronic X CIS	0.01	0.18	0.86	Chronic X CIS	0.15	2.58	0.01	Chronic X CIS	0.06	1.05	0.29
Chronic X RR	0.17	0.85	0.39	Chronic X RR	0.26	1.41	0.16	Chronic X RR	–0.14	–0.70	0.49
Chronic X SPMS	0.30	1.42	0.16	Chronic X SPMS	0.30	1.51	0.13	Chronic X SPMS	–0.17	–0.83	0.41
Adjusted R-squared	0.00			Adjusted R-squared	0.10			Adjusted R-squared	0.00		
N	523			N	526			N	535		
Active lesion burden models											
Age	0.00	–0.09	0.93	Age	–0.18	–3.83	0.00	Age	–0.07	–1.40	0.16
Gender	–0.01	–0.33	0.74	Gender	–0.08	–1.84	0.07	Gender	–0.04	–0.88	0.38
Active Burden	–0.04	0.00	1.00	Active Burden	–11.72	–0.89	0.38	Active Burden	–4.71	–0.34	0.73
CIS	–0.09	–0.81	0.42	CIS	–0.48	–4.95	0.00	CIS	–0.10	–0.99	0.32
RR	–0.17	–1.09	0.28	RR	–0.75	–5.39	0.00	RR	–0.10	–0.67	0.50
PMS	–0.22	–1.55	0.12	PMS	–0.73	–5.59	0.00	PMS	–0.03	–0.25	0.81
Active X CIS	0.01	0.00	1.00	Active X CIS	2.81	0.9	0.37	Active X CIS	1.02	0.34	0.73
Active X RR	0.01	0.00	1.00	Active X RR	11.42	0.89	0.38	Active X RR	4.56	0.34	0.73
Active X SPMS	–0.02	–0.03	0.97	Active X SPMS	0.51	0.84	0.40	Active X SPMS	0.15	0.24	0.81
Adjusted R-squared	–0.01			Adjusted R-squared	0.09			Adjusted R-squared	0.00		
N	504			N	505			N	514		
Brain volume models											
Age	0.03	0.66	0.51	Age	–0.13	–3.12	0.00	Age	0.03	0.55	0.58
Gender	–0.02	–0.52	0.60	Gender	0.01	0.45	0.65	Gender	–0.01	–0.32	0.75
Brain Volume*	0.09	1.00	0.32	Brain Volume*	0.22	3.08	0.00	Brain Volume*	0.10	1.16	0.25
White Matter Vol.	–0.01	–0.09	0.93	White Matter Vol.	0.11	1.40	0.16	White Matter Vol.	–0.10	–1.02	0.31
CIS	0.19	0.22	0.82	CIS	0.92	1.26	0.21	CIS	0.32	0.39	0.70
RR	–0.86	–1.13	0.26	RR	0.98	1.59	0.11	RR	–0.45	–0.60	0.55
SPMS	0.72	0.97	0.33	SPMS	1.20	1.98	0.05	SPMS	–1.09	–1.50	0.13
Brain Vol X CIS	–0.31	–0.57	0.57	Brain Vol X CIS	–0.61	–1.34	0.18	Brain Vol X CIS	–0.12	–0.23	0.82
Brain Vol X RR	–0.04	–0.09	0.93	Brain Vol X RR	–1.01	–2.58	0.01	Brain Vol X RR	0.06	0.13	0.90
Brain Vol X SPMS	–0.71	–1.47	0.14	Brain Vol X SPMS	–0.78	–1.98	0.05	Brain Vol X SPMS	–0.18	–0.38	0.70
WMV X CIS	0.11	0.16	0.87	WMV X CIS	–0.60	–0.97	0.33	WMV X CIS	–0.25	–0.40	0.69
WMV X RR	0.86	1.25	0.21	WMV X RR	–0.44	–0.78	0.44	WMV X RR	0.35	0.52	0.61
WMV X SPMS	–0.12	–0.20	0.84	WMV X SPMS	–0.77	–1.60	0.11	WMV X SPMS	1.25	2.19	0.03
Adjusted R-squared	0.01			Adjusted R-squared	0.28			Adjusted R-squared	0.00		
N	732			N	759			N	757		
Subcortical gray matter volume models											
Age	0.03	0.74	0.46	Age	–0.15	–4.11	0.00	Age	–0.01	–0.19	0.85
Gender	–0.02	–0.43	0.67	Gender	0.02	0.70	0.49	Gender	–0.01	–0.38	0.71
Subcortical Health*	0.12	1.10	0.27	Subcortical Health*	0.35	4.01	0.00	Subcortical Health*	0.01	0.11	0.91
Amygdala Vol.	–0.02	–0.27	0.78	Amygdala Vol.	–0.16	–2.39	0.02	Amygdala Vol.	0.06	0.81	0.42
CIS	0.00	–0.01	1.00	CIS	0.60	1.26	0.21	CIS	–0.27	–0.45	0.65
RR	–0.27	–0.47	0.64	RR	0.37	0.81	0.42	RR	–0.03	–0.05	0.96
SPMS	0.37	0.72	0.47	SPMS	0.48	1.18	0.24	SPMS	0.05	0.11	0.91
Subcortical X CIS	0.04	0.06	0.95	Subcortical X CIS	–1.03	–2.07	0.04	Subcortical X CIS	0.22	0.37	0.71
Subcortical X RR	0.16	0.27	0.79	Subcortical X RR	–1.57	–3.43	0.00	Subcortical X RR	0.24	0.44	0.66
Subcortical X SPMS	–0.64	–1.26	0.21	Subcortical X SPMS	–1.29	–3.12	0.00	Subcortical X SPMS	–0.03	–0.06	0.95
Amygdala X CIS	–0.06	–0.17	0.87	Amygdala X CIS	0.15	0.52	0.61	Amygdala X CIS	–0.01	–0.04	0.97
Amygdala X RR	0.08	0.22	0.83	Amygdala X RR	0.77	2.70	0.01	Amygdala X RR	–0.27	–0.79	0.43
Amygdala X SPMS	0.19	0.58	0.56	Amygdala X SPMS	0.48	1.83	0.07	Amygdala X SPMS	–0.04	–0.12	0.90
Adjusted R-squared	0.01			Adjusted R-squared	0.29			Adjusted R-squared	–0.01		
N	731			N	758			N	756		

*CIS*, Clinically Isolated Syndrome, *RRMS* Relapsing Remitting MS, *SPMS* Secondary Progressive MS

^a^Interaction terms are denoted by “X’ between main effect terms in the bottom section of each table. The referent group is healthy controls

### Relapsing-remitting group-specific analyses of MRI reserve metrics

**Fig. 1:**
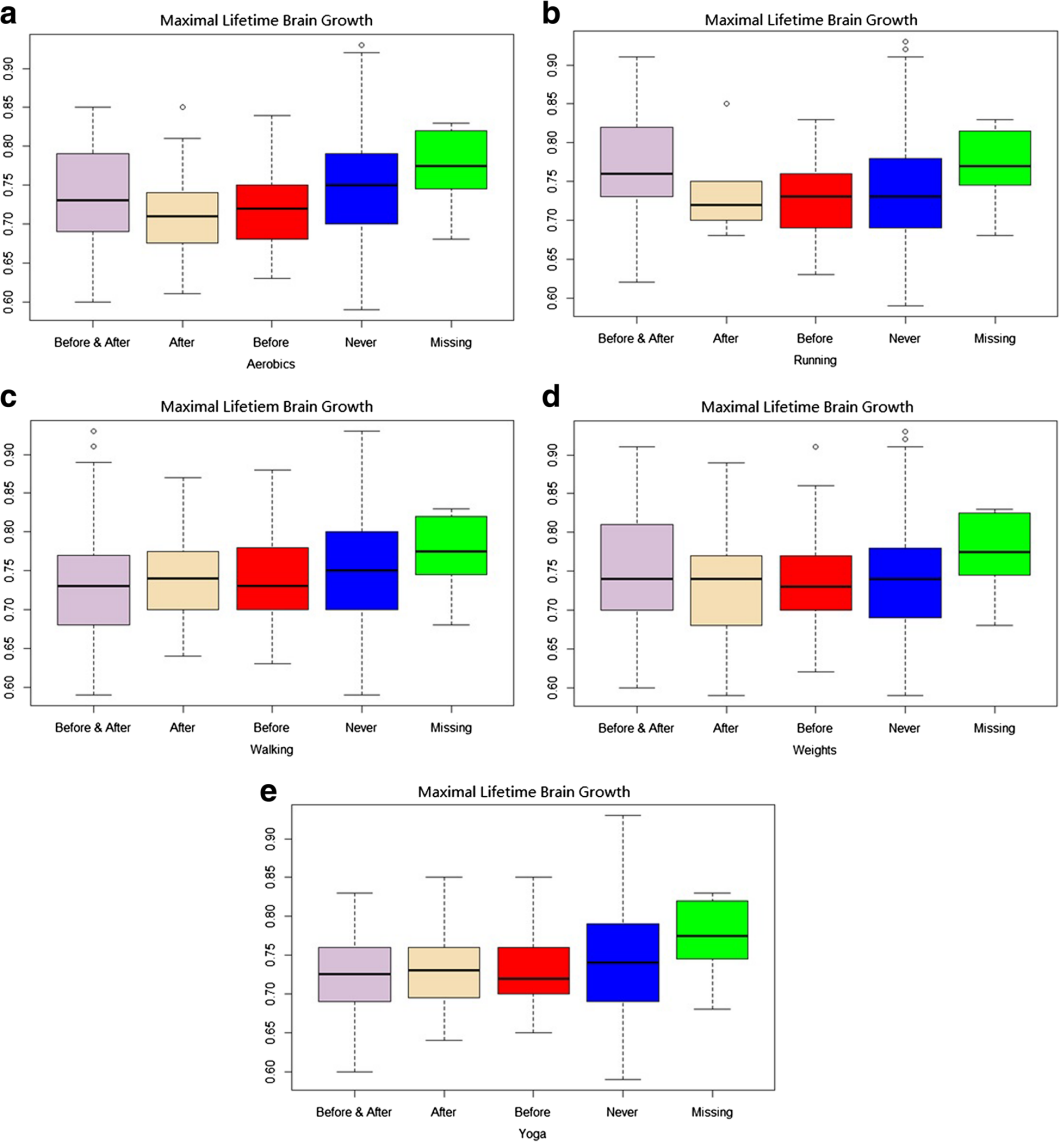
**a**-**e** Box plots of Maximal Lifetime Brain Growth in RRMS patients. Shown by change in strenuous activity group for aerobics, running, walking, weights, and yoga. On the far left is the box plot for individuals who engaged in the strenuous activity both before and after MS diagnosis. The box plot immediately to the right is for those individuals who engaged only after, then only before, then never. The far right box plot show the distribution on the Maximal Lifetime Brain Growth score for those individuals missing data on that activity

Figures 1a-e show box plots of Maximal Lifetime Brain Growth (maximum lifetime brain growth) by change in strenuous activity group for aerobics, running, walking, weights, and yoga. On the far left is the box plot for individuals who engaged in the strenuous activity both before and after MS diagnosis. The box plot immediately to the right is for those individuals who engaged only after, then only before, then never. The far right box plot show the distribution on the Maximal Lifetime Brain Growth score for those individuals missing data on that activity.

There were significant differences between groups on aerobics (Kruskal Wallis χ^2^ = 14.57, *p =* 0.01), such that the Never group differed from the Only-After and Only-Before groups and the Missing group differed from all but the Never groups (Additional file 2: Table S2). There were significant differences between groups on running (Kruskal Wallis χ^2^ = 11.77,*p =* 0.02), such that the Before-and-After Group differed from the Only-Before and Never groups, and the Missing group differed from the Only-Before and Never groups (Additional file 2: Table S2). The non-parametric comparisons for yoga showed trend significance (Kruskal Wallis χ^2^ = 8.69, *p =* 0.07), with the differences being between Before-and-After and Only-after versus Missing. There were no significant differences in the Walking or Weights change groups with respect to Maximal Lifetime Brain Growth.

**Fig. 2:**
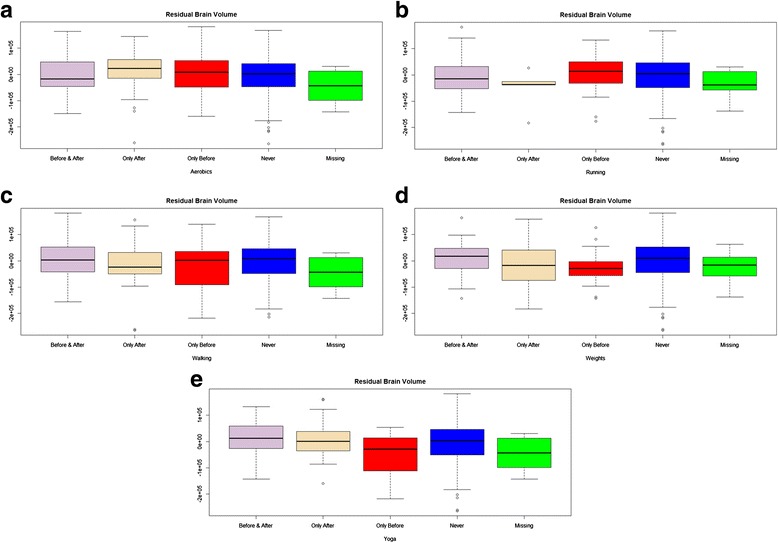
**a**-**e** Box plots of Residual Brain Volume in RRMS patients. Shown by change in strenuous activity group for aerobics, running, walking, weights, and yoga. On the far left is the box plot for individuals who engaged in the strenuous activity both before and after MS diagnosis. The box plot immediately to the right is for those individuals who engaged only after, then only before, then never. The far right box plot show the distribution on the Residual Brain Volume score for those individuals missing data on that activity

Figures 2a-e show box plots of the Residual Brain Volume by change in strenuous- activity group for aerobics, running, walking, weights, and yoga, set up identically to Fig. 1a-e. There was a significant difference between groups on weights (Kruskal Wallis χ^2^ = 10.8,*p =* 0.03), such that the Only-Before group differed from the Before-and-After and the Never groups (Additional file 2: Table S2). There were no significant differences in any of the other strenuous change groups and Residual Brain Volume.

## Discussion

Our findings suggest that MRI metrics related to chronic lesion burden, brain volume, and subcortical gray matter volume were differentially associated with MS groups in predicting strenuous reserve-related activities, but not for past activities or for current non-strenuous activities. A closer look at changes in strenuous activities over time in RR patients suggests that among those individuals who provided data on these variables, people who engaged in running before and after diagnosis, rather than only before or never, had higher Maximal Lifetime Brain Growth. That is, their current activity is *associated with* pre-morbid brain size. People who engaged in aerobics only after or only before differed from those who never engaged in aerobics. Among those individuals who provided data on these variables, individuals who engaged in weights-related exercise before and after diagnosis, as compared to only before or never had lower Residual Brain Volume. These findings suggest that reserve-related activities are related to brain health both cross-sectionally in MS subgroups, and with regard to changes in activities over time in RR patients. These findings support the importance of environmental factors in facilitating reserve-related activities and potentially buffering individuals from disease burden and progression.

There are limitations to our findings which must be considered. First, this is an observational study that investigates a research question that has not been addressed before. Accordingly, we did not have a priori hypotheses, nor did we know how many comparisons we would make a priori; we thus did not adjust for multiple comparisons in our statistical analyses. We are simply reporting on what was observed in this relatively large observational cohort study. Future research by others might examine this research question in an independent sample and evaluate whether the effects detected are small, medium, or large effect sizes. Further, because the data are cross-sectional, they may suffer from biases associated with retrospective self-report. Our ability to make causal inferences is thus hindered: the findings could either reflect a buffering effect or reverse causality—that people with worse health outcomes participate in fewer stimulating leisure activities because of their worse health. Further, the confidence intervals for the ‘never’ group in all of the plots shown in Figs. 1a-e and 2a-e are very wide, suggesting that this is a ‘noisy’ estimate which should be interpreted with caution. Additionally, we did not assess neurocognitive status or other factors associated with brain health, such as genetic contributions or trauma. In the subgroup analysis focused on RR patients, our findings were consistent with our hypothesis, suggesting that higher Maximal Lifetime Brain Growth and current reserve are associated with maintaining strenuous activities in spite of having an MS diagnosis. It is equally possible, however, that people with more aggressive MS are less likely to continue strenuous activity because of physical disability or transient heat-related symptom worsening. Additionally, the varying sample sizes by MS group and missing data result in varying statistical power for the multivariate comparisons, and possible biases in the strenuous-change analyses. We tried to counteract this challenge by using non-parametric analyses, and by explicitly comparing results on the missing-data group.

It is notable that a large number of statistical comparisons were done, while relatively few statistically significant associations were found. While this study was initiated without a priori hypotheses that would have driven specific hypothesis- testing or power calculations aimed at detecting pre-specified effect sizes, the number of comparisons done might have led to false positive findings. Rather than employing one or another conservative adjustment for the Type I error rate, we simply describe our findings. We hope that the high level of transparency we have strived for regarding the analyses done will facilitate future research aimed at replicating and confirming the findings It is also possible that the MRI metrics used here are not sensitive enough to measure plasticity. Research on brain plasticity suggests that stimulating activities are associated with structural brain changes [30]. Strenuous activities, such as aerobic exercise, have been associated with increased neurogenesis in the dentate gyrus of the hippocampus, and upregulation of brain-derived neurotrophic factor [31]. Cognitive training has been found to result in focal volume changes in areas relevant to task demand [32, 33]. Barulli and Stern [2] suggest that these findings support the long-term benefits of intellectual stimulation and physical exercise, and that such exposures may not only help the brain to adapt to structural changes, but also may help to prevent those changes to begin with. Future research might use connectome structural and functional MRI measures to address reserve.

Despite the above limitations, the study’s strengths are substantial: it has a large sample with MRI data, includes healthy controls as a reference group, utilizes novel MRI metrics, and captures exercise patterns before and after MS diagnosis to allow an evaluation of the its impact on brain health.

## Conclusions

Our findings are suggestive that MRI metrics relate to reserve-related activities, although our cross-sectional design renders our findings somewhat unclear with regard to direction of association in the correlations or multivariate models. Prospective research is needed to definitively support the buffering effect of reserve-related activities, but we believe that the findings of the present study build on a growing evidence base that should encourage people with MS to maintain their engagement in a range of strenuous stimulating activities to promote health and prevent disability progression.

## Abbreviations

CIS, clinically isolated syndrome; EDSS, expanded disability status scale; ICV, intracranial volume; MRI, magnetic resonance imagine; MS, multiple sclerosis; RR, relapsing-remitting; SPMS, secondary progressive multiple sclerosis

## Additional files

Additional file 1: Table S1. Descriptive statistics of MRI measures. (XLS 10 kb) Additional file 2: Table S2. Results of non-parametric tests comparing strenuous change groups in relapsing-remitting patients. (XLS 13 kb)
